# Volatile Oils Discrepancy between Male and Female *Ochradenus arabicus* and Their Allelopathic Activity on *Dactyloctenium aegyptium*

**DOI:** 10.3390/plants12010110

**Published:** 2022-12-26

**Authors:** Ahmed M. Abd-ElGawad, Abdulaziz M. Assaeed, Abd El-Nasser G. El Gendy, Basharat A. Dar, Abdelsamed I. Elshamy

**Affiliations:** 1 Plant Production Department, College of Food & Agriculture Sciences, King Saud University, P.O. Box 2460, Riyadh 11451, Saudi Arabia; 2 Department of Botany, Faculty of Science, Mansoura University, Mansoura 35516, Egypt; 3 Medicinal and Aromatic Plants Research Department, National Research Centre, Cairo 11865, Egypt; 4 Department of Natural Compounds Chemistry, National Research Centre, 33 El Bohouth St., Dokki, Giza 12622, Egypt

**Keywords:** *Ochradenus arabicus*, essential oils, phytotoxicity, weed control, isothiocyanates

## Abstract

Volatile oils (VOs) composition of plants is affected by several exogenous and endogenous factors. Male and female plants of the dioecious species exhibit variation in the bioactive constituents’ allocation. The chemical variation in the VOs between male and female plants is not well studied. In the present study, the chemical characterization of the VOs extracted from aerial parts of male and female ecospecies of *Ochradenus arabicus* was documented. Additionally, the extracted VOs were tested for their allelopathic activity against the weed *Dactyloctenium aegyptium*. Via GC-MS analysis, a total of 53 compounds were identified in both male and female plants. Among them, 49 compounds were identified from male plants, and 47 compounds were characterized in female plants. Isothiocyanates (47.50% in male and 84.32% in female) and terpenes (48.05% in male and 13.22% in female) were the main components of VOs, in addition to traces of carotenoid-derived compounds and hydrocarbons. The major identified compounds of male and female plants are *m*-tolyl isothiocyanate, benzyl isothiocyanate, butyl isothiocyanate, isobutyl isothiocyanate, carvone, and α-bisabolol, where they showed variation in the concentration between male and female plants. The *O. arabicus* VOs of the male plants attained IC_50_ values of 51.1, 58.1, and 41.9 μL L^−1^ for the seed germination, seedling shoot growth, and seedling root growth of the weed (*D. aegyptium*), respectively, while the females showed IC_50_ values of 56.7, 63.9, and 40.7 μL L^−1^, respectively. The present data revealed that VOs composition and bioactivity varied significantly with respect to the plant gender, either qualitatively or quantitatively.

## 1. Introduction

The plant kingdom encompasses about 320,000 known species that are very rich resources of metabolites, which play crucial roles in the growth, reproduction, and defense mechanism of plants [1]. A myriad of metabolites with potent bioactivities was reported from the plants including essential and/or volatile oils [2]. The volatile oils (VOs) derived from the plants are mixtures of volatile low molecular weights constituents extracted by several extraction techniques. These compounds are categorized under various classes such as mono-, sesqui-, and di-terpenes carotenoid-derived compounds, apo-carotenoid-derived compounds, phenylpropanoids, and other hydrocarbons [3,4]. The VOs were reported to exert several biological and pharmaceutical potentialities such as antiviral [5], antimicrobial [6], anticancer [7,8], anti-inflammatory [9,10], antipyretic [9,11], and antiulcer [12] effects.

The VOs of plants are affected by several external factors including environmental factors, such as temperature, light, moisture, atmospheric oxygen, precipitation, soil characteristics [13,14], geographic variations [15], seasonal variation, and climatic factors [16]. In addition, VOs can be affected by endogenous factors such as developmental stages [17], genetic variability [18,19], and variety [20]. Moreover, plant sex has been reported to affect the chemical composition of the VOs either quantitively or qualitatively in many plants, such as *Laurus nobilis* L. [21] and *Juniperus communis* L. [22].

The plants belonging to the *Ochradenus* genus (Family: Resedaceae), including around eight species, are widely distributed in Southwest Asia, North Africa, and the Arabian Peninsula [23]. *Ochradenus arabicus* Chaudhary, Hillc. & A.G.Mill. is a compact twiggy dioecious shrub (up to 75 cm tall) with small yellow flowers as well as yellow, papery fruits [24]. The male and female reproductive organs are present in separate individuals. *O. arabicus* is a plant endemic to the Arabian Peninsula, where it is reported only in the flora of Oman, Saudi Arabia, Yemen (Soqotra), and the United Arab Emirates. This shrub is one of the most common medicinal plants with several significant bioactivities such as antioxidant, anticancer, antimicrobial, antidiabetic, anti-indole acetic acid genotoxicity, and allopathic [25,26,27]. The documented phytochemical studies of the various extracts of *O. arabicus* revealed the identification of phenolic compounds including several flavonoids [25,26]. Recently, the aroma profiling, antioxidant, antimicrobial, and antidiabetic effects of VOs derived from the different organs of the *O. arabicus* collected from Oman were documented [28]. Although *O. arabicus* is a dioecious plant, in this study, the authors did not explain if they targeted the male or female plant of *O. arabicus*. Additionally, we hypothesized that the chemical composition of the VOs of *O. arabicus* would be varied according to the sex of the plant. Thereby, the objectives of the current work were (1) to assess the variation in the chemical composition of the VOs derived from the aerial parts of the male and female plants of *O. arabicus* collected from Saudi Arabia, and (2) to evaluate the allelopathic activity of the extracted VOs from male and female plants.

## 2. Results and Discussion

### 2.1. Male and Female Ochradenus arabicus VOs Chemical Profiling

**Figure 1:**
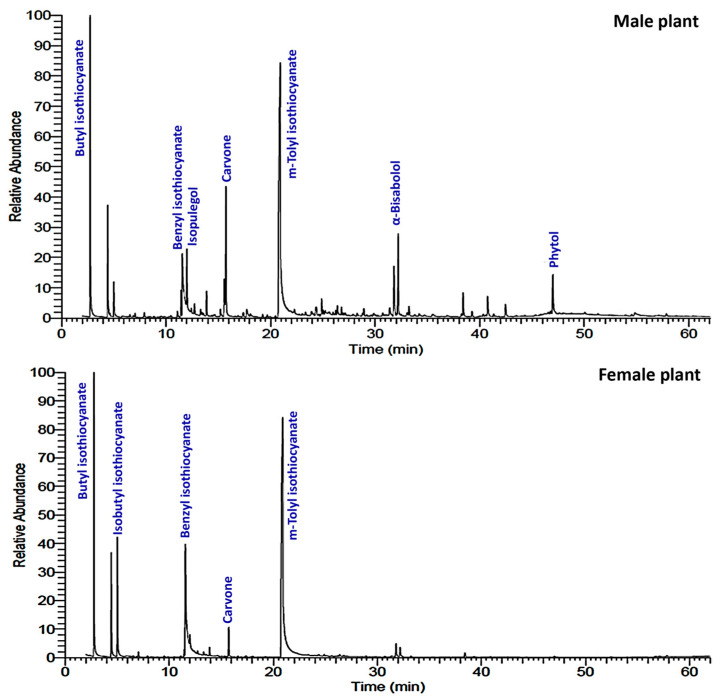
GC-MS chromatograms of male and female *Ochradenus arabicus* volatile oils.

**Figure 2:**
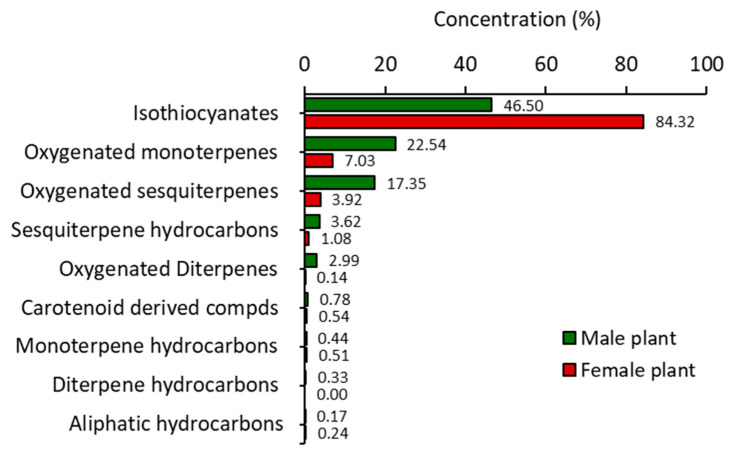
Various classes of the identified chemical compounds in male and female plants of *Ochradenus arabicus*.

The air-dried samples of male and female *O. arabicus* were subjected to hydrodistillation for three hours over the Clevenger apparatus, where they produced 0.08% and 0.07% (*v*/*w*) of the pale-yellow VOs, respectively. The analysis of the VOs samples was performed by the GC-MS (Figure 1). The chemical profiles were summarized in Table 1 including the Rt (retention times), literature, and experimental KI (retention indices), along with relative concentrations. The data revealed that VOs’ profiles of male and female *O. arabicus* included nine classes of components (Figure 2). The female plants were rich in isothiocyanates (84.32%) compared to the male plants (46.50%). However, male plants were richer in terpenoid compounds (47.27), compared to female plants (12.68%). The majority of the terpenoid classes are oxygenated monoterpenes and oxygenated sesquiterpenes in both male and female *O. arabicus* (Figure 2). Generally, the oxygenated compounds were higher in male (43.27%) than female plants (11.20%), while the non-oxygenated compounds were identified in low concentration in both male (4.39%) and female plants (2.31%). The abundance of the oxygenated compounds was already reported in the previous analysis of EO derived from different organs of *O. arabicus* collected from Oman but without specification of the gender [28].

**Figure 3:**
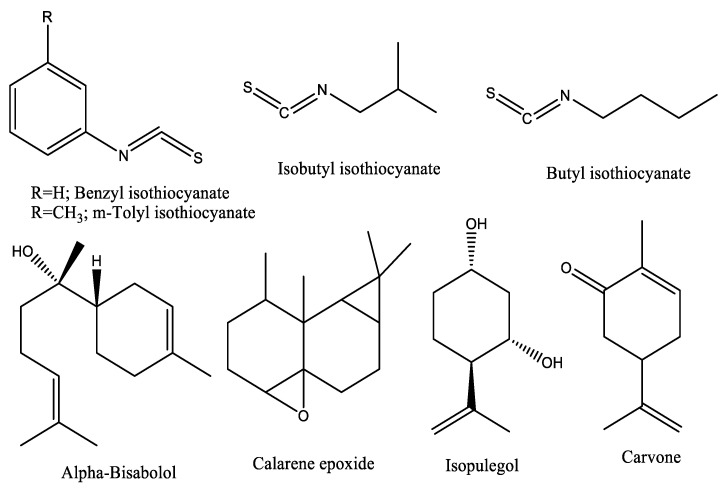
Chemical structures of the main identified compounds in the volatile oils of *Ochradenus arabicus*.

Overall, 53 compounds were identified in the VOs of both male and female *O. arabicus*. Forty-nine compounds (94.72% of the total mass) were identified in the VO of the male plant, while 47 compounds, with a total relative concentration of 97.78%, were assigned from the female plant. The isothiocyanates were assigned as the major constituents of both genders, while the female was richer than the male plants. Four isothiocyanates were characterized with high relative concentrations in male and female ecospecies including *m*-tolyl isothiocyanate (35.3 and 55.41%), benzyl isothiocyanate (4.88 and 14.08%), butyl isothiocyanate (4.77 and 6.84%), and isobutyl isothiocyanate (1.55 and 7.99%) (Figure 3).

The abundance of isothiocyanates in the present study is not in agreement with previously documented data on the VOs of the stems, leaves, and flowers of *O. arabicus* collected from Oman [28]. This wide variation between our data and that identified in the Omani *O. arabicus* can be ascribed to the extraction technique, where Ullah, et al. [28] made the extraction by heating the Clevenger machine for a long time till no further oil was extracted. Chen and Ho [29] described that isothiocyanate is easily decomposed with refluxing for one hour at 100 °C. Additionally, De Nicola, et al. [30] deduced that the benzylic-Isothiocyanates are unstable and easily converted to other derivatives after refluxing at 90 °C. Thereby, the absence of isothiocyanates in the data of the Omani *O. arabicus* could be attributed to the degradation of the isothiocyanate due to the extraction process.

The isothiocyanates were characterized as main volatiles in the VOs of several plants such as *Wasabia japonica* (Miq.) Koidz. [31]; *Brassica oleracea* L.; *B. rapa* L.; *Armoracia lapathifolia* G. Gaertn., B. Mey. & Scherb.; *Eutrema japonicum* (Miq.) Koidz.; and *Carica papaya* L. [32].

Terpenoids represented the main constituents of the male *O. arabicus*, with a higher relative concentration than the female plant. Six classes of terpenes were characterized from the male plant comprising monoterpene hydrocarbons (0.44%), oxygenated monoterpenes (22.54%), sesquiterpene hydrocarbons (3.62%), oxygenated sesquiterpene (17.35%), diterpene hydrocarbons (0.33%), and oxygenated diterpenes (2.99%). Five terpene classes were assigned from the female plant including monoterpene hydrocarbons (0.51%), oxygenated monoterpenes (7.03%), sesquiterpene hydrocarbons (1.08%), oxygenated sesquiterpenes (3.92%), and oxygenated diterpenes (0.14%) (Figure 2). The present data are in harmony with the data published for Omani *O. arabicus* [28].

Carvone (7.80%), α-bisabolol (5.77%), calarene epoxide (3.79%), isopulegol (3.49%) (Figure 3), *trans*-chrysanthenyl acetate (3.02), and phytol (2.99%) were found as the fundamental components of the VO of the male plant. Furthermore, carvone (3.01%), calarene epoxide (1.68%), and α-bisabolol (1.11%) were the major constituents of the female plants’ VO. The diversity of terpenoids in the current findings was in harmony with previously reported data of VO derived from *O. arabicus* collected from Oman [28]. However, the composition of the VO profile is different, i.e., the major compounds of the current study varied from those reported for the Omani ecospecies. Carvone as the main compound of the current study was reported as the main constituent of several plants such as *Tanacetum balsamita* L. [33], *Mentha longifolia* (L.) Huds., and *M. spicata* L. [34]. Furthermore, the major compound in the present study, α-bisabolol, was a common major compound in EOs of numerous plants such as *Matricaria chamomilla* L., *Salvia runcinata* L.f., *Smyrniopsis aucheri* Boiss., *Eremanthus erythropappus* (DC.) MacLeish, and other *Vanillosmopsis* Sch.Bip. species [35].

Finally, traces of the carotenoid-derived compounds, represented by two compounds, theaspirane B and α-ionone, were identified in the VOs of both male and female *O. arabicus*. In addition, one hydrocarbon compound, *n*-tricosane (0.17%), was identified in VOs of the male plants while two hydrocarbons, *n*-docosane (0.08) and *n*-tricosane (0.16%), were assigned from female plants. The tricosane was reported in trace amounts (0.53%) in the leaf VOs of the *O. arabicus* collected from Oman [28].

The significant variation in the chemical constituents between the current results and previous data might be ascribed to variations in organs, genotypes, ages, climate, weather, humidity, and environmental conditions [36,37,38]. Moreover, our data revealed the role of the gender of the plant on the phytochemical compositions including the VOs [39,40]. The richness of the compounds in the male plants compared to the female ones could be attributed to the fact that female plants invest less in chemical defense and more into biomass production than male plants [41], where the VOs can be considered a good indicator for the degree of chemical defense in plants due to its distinct variability with the biotic and abiotic stresses [22,42].

### 2.2. Chemometric Analysis of the VOs from Male and Female O. arabicus

**Figure 4:**
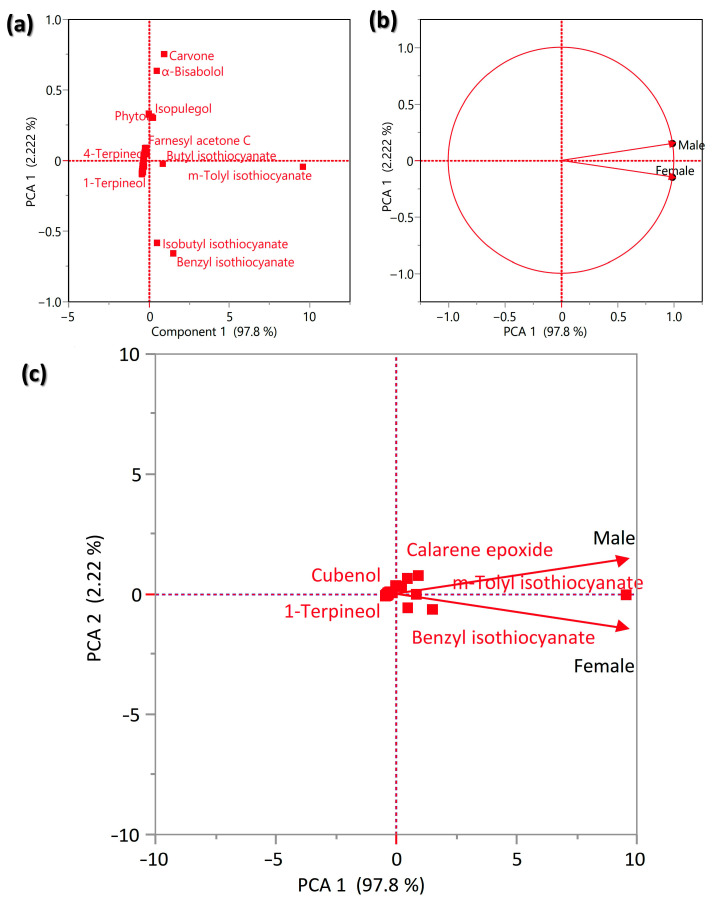
The Principal Components Analysis (PCA) of the identified volatile compounds in male and female *O. arabicus*. (**a**) The observation in the PCA space, (**b**) correlation circle (variables chart), and (**c**) biplot.

In order to show the variation between male and female plants of *O. arabicus*, the data of the concentration of all identified compounds in the VOs were subjected to the Principal Components Analysis (PCA), where it revealed a slight variation in the composition (Figure 4). The main components that showed clear segregation were isobutyl isothiocyanate, benzyl isothiocyanate, m-tolyl isothiocyanate, butyl isothiocyanate, farnesyl acetone C, isopulegol, phytol, α -bisabolol, and carvone (Figure 4a). The male plants showed a close correlation with m-tolyl isothiocyanate and calarene epoxide, while female plants showed a close correlation with benzyl isothiocyanate and m-tolyl isothiocyanate (Figure 4c). This variation between male and female plants supports the issue that a plant’s gender affects the chemical composition of the secondary metabolites [21,40].

### 2.3. Allelopathic Activity of Male and Female O. arabicus VOs on the Weed D. aegyptium

**Figure 5:**
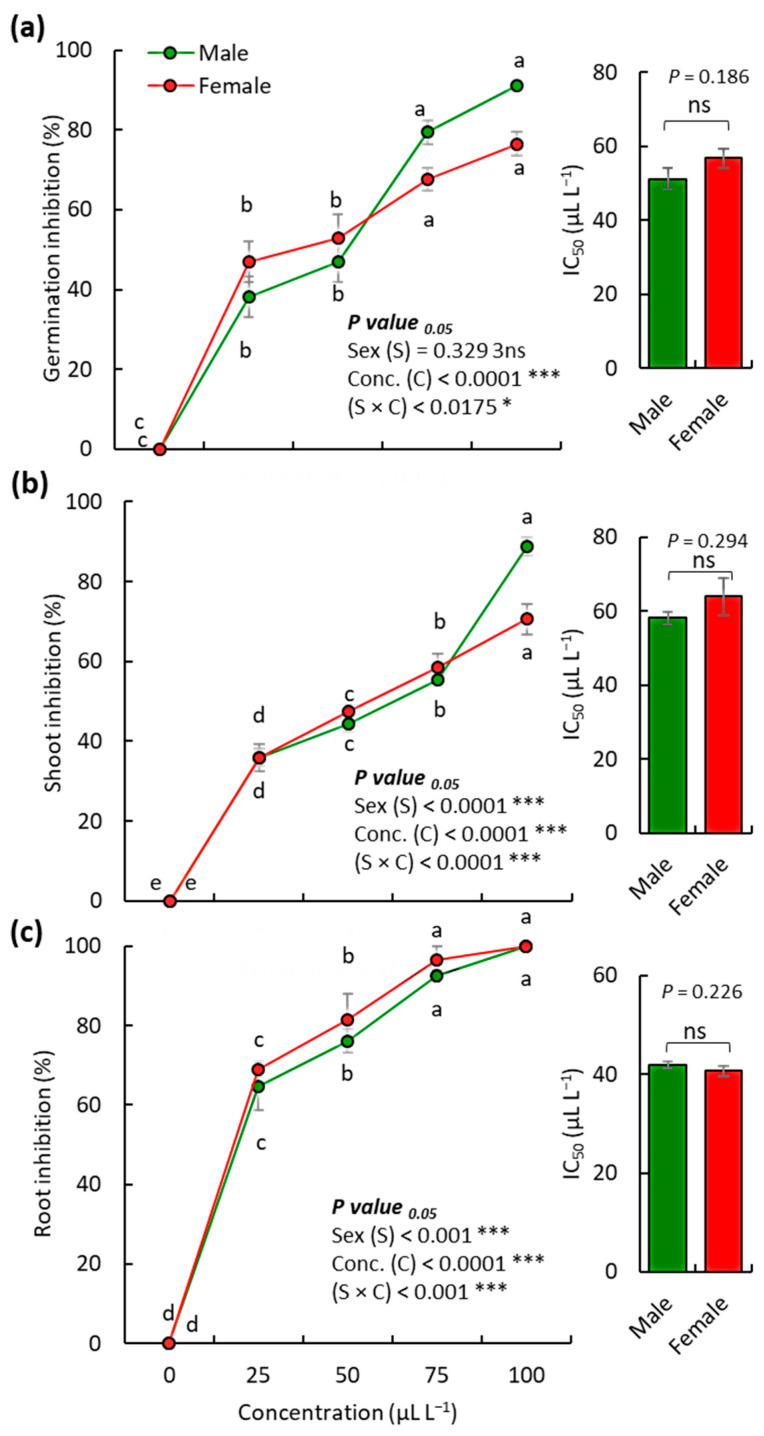
Allelopathic activity of various concentrations of the extracted volatile compounds of male and female *O. arabicus* against the seed germination (**a**), shoot growth (**b**), and root growth (**c**) of *D. aegyptium*. * *p* ˂ 0.05, *** *p* ˂ 0.001, and “ns” for *p* > 0.05.

The VOs extracted from both male and female plants of *O. arabicus* showed significant allelopathic activity against the weed *Dactyloctenium aegyptium* (L.) Willd in a dose-dependent trend (Figure 5). The highest concentration of the VOs (100 μL L^−1^) showed inhibition of *D. aegyptium* germination by 91.18% and 76.47% for male and female plants, respectively (Figure 5a). The root growth of *D. aegyptium* seedlings was more affected by the VOs compared to the shoot; this could be ascribed to direct contact with the VOs in the medium as well as the permeability of root cells [38,43,44]. The shoot growth of *D. aegyptium* was inhibited by 88.84% and 70.62% after treatment at a concentration of 100 μL L^−1^ of the VOs (Figure 5b). However, at a concentration of 100 μL L^−1^ of the VOs application, the seedling root growth was totally inhibited for both male and female plant extracts, while at a concentration of 75 μL L^−1^, the root growth was decreased by 96.50% and 92.42% for male and female plants, respectively (Figure 5c).

Based on the IC_50_ values, no significant variation in either the germination or seedling growth of *D. aegyptium* was observed between the male and female plants of *O. arabicus* (Figure 5). The *O. arabicus* VOs of the male plants attained IC_50_ values of 51.10, 58.05, and 63.91 μL L^−1^ for the seed germination, seedling shoot growth, and seedling root growth, respectively, while the female plants showed IC_50_ values of 56.75, 41.93, and 40.71 μL L^−1^, respectively. These data showed that the variation in the VOs composition (either quality or quantity) has a consequential effect on its biological activities [45]. In this context, the male plants of *Baccharis dracunculifolia* DC. have been reported to produce higher levels of essential oil and phenolic compounds compared to female plants, which leads to greater antioxidant capacity [46]. Moreover, the bioactive compounds (total phenolics, flavonoids, and tannins) and antioxidant activities of *Pistacia atlantica* Desf. were more influenced by growing region than by gender [47].

The major compound, m-tolyl isothiocyanate, in the present study has been reported to possess allelopathic activity against various crops such as wheat, lettuce, cowpea, and barnyard grass [48]. In addition, isothiocyanate is reported as a potential inhibitor of germination and growth in many weedy species such as *Cyperus rotundus* L. and *C. esculentus* L. [49]. Moreover, it seems that plants produce isothiocyanate compounds as a defense strategy, where these compounds showed inhibitory activity against microbial plant pathogens [50] and insects [51]. In the same context, the oxygenated monoterpene carvone (a major compound in the present study) has been described to inhibit the growth of weeds [52].

The slight variation in the allelopathic activities between male and female plants of *O. arabicus* VOs in the present study could be attributed to the variation in chemical composition [45]. It is worth mentioning here that oxygenated compounds have more biological activities compared to non-oxygenated compounds, where active groups/sites in the oxygenated compounds showed a more interactive effect [38,53]. In the present study, the oxygenated compounds in the male plants (43.27%) were higher than in female plants (11.20%), which could explain the higher allelopathic activity of the male *O. arabicus* VOs.

Comparing male and female plants of *O. arabicus*, the female plants have a higher content of isothiocyanates (76.33%) and lower content of oxygenated compounds, compared to the male plants, which have a higher content of oxygenated compounds and lower content of isothiocyanates (44.95%). This situation could explain the comparable allelopathic activity against *D. aegyptium.*

## 3. Materials and Methods

### 3.1. Plant Collection and Preparation

**Figure 6:**
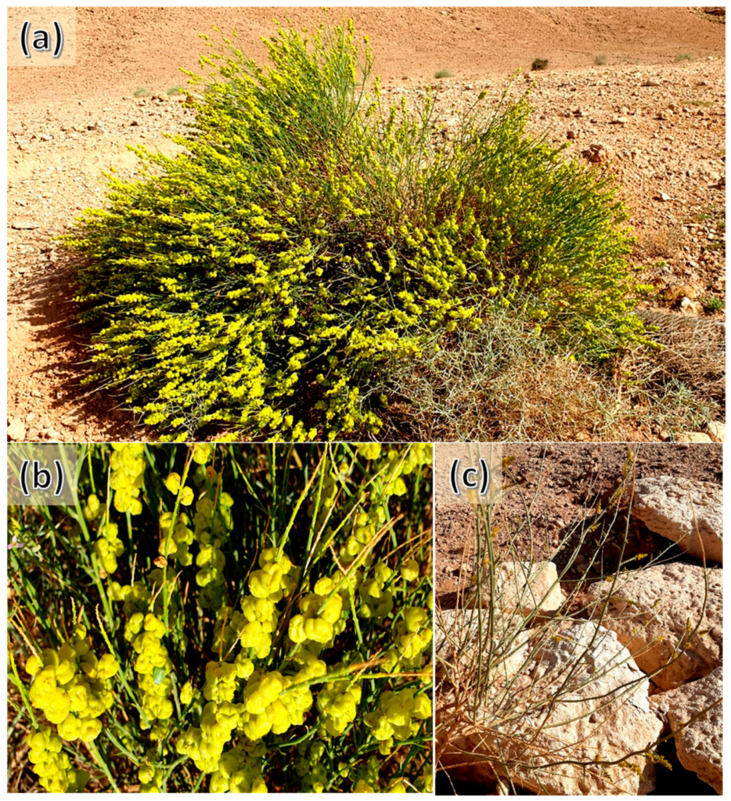
*Ochradenus arabicus* Chaudhary, Hillc. & A.G.Mill. female shrub. (**a**) Overview of the female shrub, (**b**) close view of fruiting branches, and (**c**) male plant. Photos by Dr. Abulaziz Assaeed (an author).

Three samples of the aerial parts (aboveground parts) of either male or female plants of *O. arabicus* were separately collected in paper bags from different individuals (*n* = 10) growing in sandy habitats at Thadiq, 130 km northern Riyadh City (25°12′55.3″ N, 45°54′53.6″ E). The plant specimen was identified by Prof. Dr. Abdulaziz Assaeed (an author) according to flora books [24,54]. A voucher sample was prepared and deposited in the herbarium of the Plant Production Department, College of Food and Agricultural Sciences, King Saud University with ID: KSU-AGRIC-181501001 (Figure 6). The samples (about 2 kg) were air dried in a shaded place at room temperature (25 ± 3 °C) for one week, till complete dryness; crushed into powder with a grinder; then packaged in paper bags and stored in the fridge at 4 °C till further analyses.

### 3.2. Extraction of the VOs, GC–MS Analysis, and Components Identification

The VOs of the male and female plants of *O. arabicus* were extracted separately from 200 g of the air-dried plant materials. In brief, the two samples were separately subjected to a hydrodistillation process for 3 h via the Clevenger apparatus. The separation of the VOs was performed by *n*-hexane and then dried with 0.5 g anhydrous sodium sulphate. These extractions were applied to the three collected samples of either male or female plants. All the extracted VOs samples were deposited at 4 °C in glass vials till the Gas Chromatography-Mass Spectrometry (GC-MS) Analysis as well as the biological assays were performed.

The GC-MS analysis of all extracted VOs samples was performed according to previously documented conditions [9,11]. The chemical components’ identification and authentication were carried out depending upon AMDIS software (Automated Mass spectral Deconvolution and Identification), NIST database, Wiley spectral collection, and *n*-alkanes (C_8_–C_22_) retention indices.

### 3.3. Allelopathic Activity Bioassay

To determine the allelopathic activity of the extracted VOs, various concentrations of the VOs were prepared and tested on the germination and seedling growth of the weed *D. aegyptium*. In brief, concentrations of 0, 25, 50, 75, and 100 μL L^−1^ of the VOs were prepared using 1% of Tween 80^®^ (Sigma-Aldrich, Darmstadt, Germany). The seeds of the targeted weed (*D. aegyptium*) were collected from the infested field and sterilized with 1% sodium hypochlorite for 3 min, followed by washing with distilled water three times, and they were then dried in air and stored in glass vials till further analysis. In Petri plates, 20 seeds of *D. aegyptium* were lined over filter paper (Whatman Grade 1), which was moistened with 5 mL of each concentration. To avoid leakage of the VOs, the Petri plates were sealed with a tape of Parafilm^®^ (Sigma, St. Louis, MO, USA). A control of Tween 80^®^ (1%) was performed with the same procedures as the treatments. A total of 90 plates [5 treatments (4 concentrations + 1 control) × 3 replications × 3 experiment times × gender (male and female plants)] were prepared and then incubated in a growth chamber adjusted at 25 ± 2 °C with a light cycle of 12 h light/12 h dark. The germination of seeds was observed and counted daily, where the seed was counted as germinated when the radicle sprouted with a 2 mm length. After 10 days of treatment, the number of germinated seeds as well as the lengths of seedling radicles and shoots were measured in mm. The inhibition of seed germination or seedling growth was calculated upon the following equation:

$$A=100\times \left\{\frac{No./Length_{control}-No./treatment_{control}}{No./Length_{control}}\right\}$$

The experiment was repeated three times with three replications for each treatment and control, and the average ± standard errors were calculated by MS-EXCEL 2019.

### 3.4. Statistical Analaysis

The data of the allelopathic activity experiment in triplicates were subjected to two-way ANOVA with gender as the first factor and concentration of the extract as the second factor at a probability level of 0.05. The analysis was followed by Tukey’s HSD test using the CoStat software program, version 6.311 (CoHort Software, Monterey, CA, USA). In addition, to test the significant variation between male and female plants, the IC50 values were subjected to a two-tailed *t*-test. On the other hand, the dataset of the concentration (%) of all identified compounds in both VOs of male and female plants of *O. arabicus* was prepared and subjected to principal component analysis (PCA) using JMP^®^ Pro 16.0.0, SAS Institute Inc., Cary, NC, USA.

## 4. Conclusions

The present study revealed for the first time substantial variations in the chemical profile of the VOs between male and female plants of *O. arabicus*, either in quantity or quality of the chemical compounds. The *m*-tolyl isothiocyanate, benzyl isothiocyanate, butyl isothiocyanate, isobutyl isothiocyanate, carvone, and α-bisabolol were the major constituents in both genders taking into consideration the differences in their relative concentrations. These data varied from those reported for Omani *O. arabicus*, although the gender was not clarified in that study. These findings support that plant gender has a significant effect on secondary metabolites in plants, coupled with environmental, climatic, and genetic factors. The extracted VOs from the two genders were found to exhibit significant allelopathic effects via the suppression of seed germination and shoot and root growth of the weed *D. aegyptium*. Furthermore, a slight difference in allelopathic activity was determined in the present study between male and female plants. This activity could be ascribed to the higher content of isothiocyanates in female plants compared to male, while male plants attained high content of oxygenated terpenes, particularly carvone and *α*-bisabolol, compared to female.

## Figures and Tables

**Table 1:** Volatile oil components of male and female plants of *Ochradenus arabicus*.

No	Compound Name	Rt ^1^	Conc. % ^2^		KI	
			Male	Female	Lit. ^3^	Exp. ^4^
	**Monoterpene hydrocarbons**					
1	α-Terpinene	6.55	0.15 ± 0.01	0.09 ± 0.01	1014	1015
2	γ-Terpinene	7.92	0.29 ± 0.02	0.42 ± 0.02	1054	1053
	**Oxygenated Monoterpenes**					
3	Eucalyptol	6.87	0.11 ± 0.01	0.05 ± 0.00	1026	1024
4	Linalool	7.05	0.18 ± 0.01	0.04 ± 0.00	1096	1097
5	1-Terpineol	9.51	0.00	0.13 ± 0.01	1133	1134
6	cis-Verbenol	10.47	0.11 ± 0.01	0.14 ± 0.01	1137	1135
7	trans-Pinocarveol	11.11	0.35 ± 0.02	0.13 ± 0.01	1139	1141
8	Camphor	11.21	0.00	0.03 ± 0.00	1141	1143
9	Menthone	11.44	1.42 ± 0.06	0.56 ± 0.02	1148	1147
10	Isopulegol	12.00	3.49 ± 0.09	1.10 ± 0.05	1149	1150
11	4-Terpineol	12.73	0.64 ± 0.03	0.00	1177	1175
12	α-Terpineol	13.44	0.58 ± 0.02	0.42 ± 0.01	1188	1190
13	trans-Carveol	13.89	1.52 ± 0.06	0.95 ± 0.03	1215	1213
14	Pulegone	15.21	0.82 ± 0.03	0.00	1233	1231
15	*trans*-chrysanthenyl acetate	15.57	3.02 ± 0.08	0.11 ± 0.01	1235	1236
16	Carvone	15.74	7.80 ± 0.16	3.01 ± 0.12	1239	1241
17	Bornyl acetate	16.90	0.11 ± 0.01	0.04 ± 0.01	1254	1257
18	Thymol	17.40	0.51 ± 0.02	0.19 ± 0.01	1290	1291
19	2-Adamantanone	17.73	0.84 ± 0.03	0.04 ± 0.01	1311	1314
20	trans-sabinenehydrate acetate	24.39	1.04 ± 0.05	0.09 ± 0.01	1577	1574
	**Sesquiterpene hydrocarbons**					
21	α-Cubebene	19.25	0.23 ± 0.01	0.10 ± 0.01	1351	1353
22	α-Ylangene	20.47	0.12 ± 0.01	0.07 ± 0.00	1373	1371
23	α-Duprezianene	23.34	0.26 ± 0.01	0.14 ± 0.01	1387	1384
24	Davana ether-1	24.90	1.16 ± 0.09	0.23 ± 0.01	1433	1430
25	Spirolepechinene	25.22	0.36 ± 0.02	0.08 ± 0.00	1451	1449
26	Dihydro-β-agarofuran	25.99	0.22 ± 0.01	0.06 ± 0.00	1503	1505
27	γ-Cadinene	26.22	0.32 ± 0.02	0.09 ± 0.01	1513	1515
28	α-Cadinene	26.39	0.73 ± 0.02	0.26 ± 0.01	1537	1539
29	α-Cadinene	27.14	0.22 ± 0.01	0.05 ± 0.00	1538	1535
	**Oxygenated Sesquiterpenes**					
30	Widdrol hydroxyether	19.94	0.00	0.06 ± 0.00	1479	1480
31	6-epi-shyobunol	26.97	0.79 ± 0.02	0.21 ± 0.01	1517	1516
32	*E*-Nerolidol	28.27	0.87 ± 0.02	0.00	1563	1560
33	Spathulenol	28.81	0.28 ± 0.01	0.04 ± 0.00	1578	1590
34	Caryophyllene oxide	28.93	0.56 ± 0.02	0.11 ± 0.00	1583	1581
35	Davanone	29.65	0.41 ± 0.01	0.13 ± 0.01	1587	1585
36	Cubenol	31.39	1.17 ± 0.06	0.23 ± 0.01	1646	1645
37	Calarene epoxide	31.81	3.79 ± 0.08	1.68 ± 0.08	1671	1670
38	α-Bisabolol	32.21	5.77 ± 0.11	1.11 ± 0.07	1685	1687
39	epi-Nootkatol	32.48	0.09 ± 0.01	0.00	1699	1601
40	Juniper camphor	33.07	0.37 ± 0.01	0.00	1700	1703
41	Drimenol	33.24	0.67 ± 0.03	0.13 ± 0.01	1767	1768
42	Hexahydrofarnesyl acetone	38.42	1.58 ± 0.07	0.54 ± 0.02	1845	1843
43	Farnesyl acetone C	40.76	1.29 ± 0.06	0.11 ± 0.01	1921	1924
	**Diterpene hydrocarbons**					
44	Cembrene	36.89	0.33 ± 0.02	0.00	1937	1939
	**Oxygenated Diterpenes**					
45	Phytol	46.98	2.99 ± 0.08	0.14 ± 0.01	1942	1945
	**Carotenoid derived compounds**					
46	Theaspirane B	18.08	0.21 ± 0.01	0.07 ± 0.00	1302	1300
47	α-Ionone	25.04	0.28 ± 0.02	0.04 ± 0.00	1430	1432
	**Isothiocyanates**					
48	Butyl isothiocyanate	4.43	4.77 ± 0.10	6.84 ± 0.13	943	941
49	Isobutyl isothiocyanate	5.01	1.55 ± 0.05	7.99 ± 0.17	978	976
50	Benzyl isothiocyanate	11.56	4.88 ± 0.07	14.08 ± 0.26	1367	1369
51	*m*-Tolyl isothiocyanate	20.93	35.30 ± 0.33	55.41 ± 0.46	1970	1972
	**Aliphatic hydrocarbons**					
52	*n*-Docosane	52.46	0.00	0.08 ± 0.00	2200	2200
53	*n*-Tricosane	57.84	0.17 ± 0.01	0.16 ± 0.01	2300	2300
	Total		94.72	97.78		

^1^ Rt: Retention time; ^2^ values are average ± SD, ^3^ KI_exp_: experimental Kovats retention index; ^4^ KI_lit_: Kovats retention index on DB-5 column with reference to n-alkanes.

## Data Availability

Not applicable.
